# The importance of front-of-pack labels in improving health status and eating behavior

**DOI:** 10.1007/s40519-023-01540-9

**Published:** 2023-02-23

**Authors:** Luca Muzzioli, Edoardo Mocini, Alessandro Pinto

**Affiliations:** Department of Experimental Medicine, Sapienza University, 00185 Rome, Italy

**Keywords:** Front-of-pack, FOPL, Food labels, Healthy and sustainable diets

## Abstract

The aim of this Editorial is to give an overview on the multiple aspects of front-of-pack labels (FOPLs) and provide the readers a balanced view of the problems raised in this research field, placing them in a wider context. Furthermore, this editorial paper discusses whether and how the use of FOPLs can promote health status in relation to the individual eating pattern and which should be the next research scenarios to investigate to further improve and integrate these tools.

Eating patterns and personal food preferences change in response to alterations in the living environment, physiological needs, way of life, and new food technologies (“from farm to fork”). People tend to consume more processed foods and proteins from animal sources while simultaneously consuming less fresh food like whole grains, legumes, vegetables, and other sources of fiber in modern society as a result of a global increase in wealth and urbanization. The changing global food supply chains are having an increasing influence on the food environment. In particular, the choice of ultra-processed foods (UPFs) in individual eating habits has increased dramatically over the past ten years due to its accessibility, decreased prices, and marketing techniques. The significant correlation between UPFs, a higher body mass index, and an increased risk of developing obesity and non-communicable diseases (NCDs) confirms that a vicious cycle has been established between individual food choices and the effects of those choices. In addition, those who are overweight or obese prefer and select more foods and beverages that are high in calories [1].

In this scenario, front-of-pack labels (FOPLs) have been created over the past decade as a tool to educate consumers about the scope of reorienting purchasing behavior, guiding individual eating patterns toward a healthy and sustainable diet, and reformulating packaged food products by the food industry. FOPLs can be divided into directive and non-directive models. Although the formers are corroborated by multiple research, the majority is carried out in simulated settings or as retrospective studies. Furthermore, directive FOPLs are referred to as simple tools, but even though they have demonstrated a high capacity for assisting consumers in classifying food products as more or less healthy, they face the risk of being assimilated to a “negative nutrition” message, removing nutrients from their context and possibly encouraging a decrease in diet variety. On the other hand, non-directive FOPLs, which are still understudied, demand cognitive effort but appear to be informative and well-liked by customers. Although they are frequently viewed as alternatives, directive and non-directive FOPLs send out different messages and both are helpful in influencing consumers’ food choices [2].

The intrinsic character of FOPLs, which, with some exceptions, typically restricts their focus to the few nutrients (i.e., the content of saturated fatty acids, sodium, or sugars) that potentially adversely affect consumers’ health status, is a key factor in the FOPLs’ debate. Information conveyed through the use of colors or numbers has, in some cases, been shown to encourage better food choices and boost the nutritional quality of the shopping basket, but it has also, in other cases, been shown to lead consumers astray and cause an incorrect assessment of the product’s healthfulness, which could lead to increased consumption of foods high in saturated fats, sodium, sugars, or calories. This is due to the fact that consumer use of FOPLs is a part of a complex framework in which the factors influencing eating and shopping behavior, such as cognitive, biological, hedonistic, and cultural factors, play a crucial role. While the purchasing scenario is defined by several stimuli that the buyer is exposed to at the point of purchase, those factors are frequently evaluated individually rather than in a synergistic context. In fact, it appears impossible to standardize an average consumer due to biases and different attitudes toward the products’ interference on purchasing behavior patterns [3].

Furthermore, focusing on specific foods and nutrients without taking the broader context of lifestyle into account is unlikely to be beneficial in promoting health and preventing disease. Numerous studies failed to discover a connection between clinical health outcomes and the consumption of foods, food groups, or single nutrients that were thought to be unfavorable or even dangerous to health. An approach based on a single nutrient or a single food has proven to be less accurate and successful than dietary pattern analysis in determining the relationship between diet and the risk of chronic diseases. Positive alterations in intermediate pathways of cardiometabolic risks, such as blood lipids, insulin sensitivity, resistance to oxidation, inflammation, and vasoreactivity, are encouraged by synergistic interactions among nutrient-rich diets. As a result, dietary pattern analysis examines the effects of the entire diet and provides greater evidence for health advantages than the impact of specific nutrients. This might be because the effects of the food matrix, which is a composite of naturally occurring food components, on human biological systems are stronger or otherwise distinct from those of the individual food components [4].

In conclusion, despite the heterogeneity of studies and approaches and the fact that most of the currently available evidence on FOPLs is based on retrospective studies, their ability to rank foods as healthy or unhealthy in accordance with the national guidelines of some countries is undisputed. Nonetheless, the actual effects of FOPLs on consumer behavior and their direct impact on individual health status and well-being are open questions. Also unresolved are their capacity to facilitate the reformulation of pre-packaged products, how to deal with traditional food products, and the actual impact on the incidence of NCDs and obesity. Given the importance of FOPLs in modern society, future research should continue to focus on their ability to improve health status and eating behaviors; on whether and how they can exacerbate or trigger eating and weight disorders, an aspect that is still understudied; and, finally, research should be directed toward the environmental impact of foods, understanding if FOPLs are capable of communicating healthiness and sustainability into a single etiquette, maintaining the simplicity of the message while embedding both environmental and dietary information.

## Data Availability

Data sharing not applicable to this article as no datasets were generated or analysed during the current study.
